# Analysis of physiological and non-contact signals to evaluate the emotional component in consumer preferences

**DOI:** 10.1371/journal.pone.0267429

**Published:** 2022-05-13

**Authors:** Rita Laureanti, Riccardo Barbieri, Luca Cerina, Luca Mainardi

**Affiliations:** Department of Electronics, Information and Bioengineering (DEIB), Politecnico di Milano, Milan, Italy; Groupe ESC Dijon Bourgogne, FRANCE

## Abstract

Emotions are an essential drive in decision making and may influence consumer preference. In this study we assessed the influence of brands in product preference after tasting 2 soft-drinks (Coca Cola vs. Cola beverage), by using physiological measurements, namely the skin conductance, the respiratory rate and heart rate variability (HRV) extracted using contactless sensors. The experimental protocol enrolled twenty-five young subjects which were asked to taste 2 soft drinks in random order, without knowing the brand (blind condition) and then knowing the brand (not blind condition). After each phase the subject was asked to choose the preferred beverage. Our main hypothesis is that if the subject knows the brand there is an arousal effect, independently from the absolute appreciation of the product. In order to evaluate the emotional components, the skin conductance, respiratory and Video-Photoplethysmographic (PPG) signals were continuously recorded throughout the experiment. The Video-PPG was then processed to extract HRV parameters. We observed that the arousal levels changed among beverages and conditions, going from higher arousal for Coca-Cola in the blind condition, to higher arousal for Cola in the not blind condition. Moreover, 44% of the subjects changed their preference when the brand was uncovered: from blind to not blind conditions, 6 subjects went from Cola to Coca-Cola as preferred drink and 5 went from Coca-Cola to Cola. Opposite results were found for the two beverages when comparing the physiological response when the beverage was/was not preferred. Finally, differences were found also between consumers and not consumers of Coca-Cola and the blind/not blind comparison. We conclude that the brand is a fundamental element in a request for choice and it can affect the first emotional response of a subject.

## Introduction

In a world always keener on consuming, companies are increasingly forced to be one step ahead of others, in particular to understand or even predict people needs. Thus, several efforts have been made to understand the various influences behind a customer choice. Since the book by Damasio in 1994, it is commonly recognized that emotion is an inseparable and central component in decision making [1]. Damasio studied patients whose ability to understand and use emotions had worsened because of brain damage (ventromedial prefrontal cortex, amygdala, somatosensory cortex) to highlight the importance of emotions and feeling in making rational choices. Such discovery evidenced the need to study the emotional response of human beings when choosing a product. Neuromarketing is a new research field born with the aim to find the tools to quantitatively assess the emotional response [2], evaluating for example the physiological response to the first instinctive response to an external stimulus. Changes in skin conductance (SC), heart rate and respiratory rate have been associated to emotions, together with the activation of specific cerebral regions. The majority of this variations can be measured either through invasive and expensive technology (such as the functional magnetic resonance imaging, fMRI), or by simpler contact technology (electrocardiogram, electroencephalogram, etc…). The contact instruments have the limitation of affecting the measure itself, both due to the direct contact to the skin or to the uncomfortable condition experienced by the subjects [3]: indeed, the subjects are limited in movements and, moreover, they can feel a guinea pig effect that affects their emotional response. For this reason, video-based contactless technologies are increasingly widespread, with the additional bonus of allowing remote measurements and reaching a larger pool of people without logistic constraints.

A particular subsection of the emotional component response is linked to the power of brand [4, 5]. McClure et al. demonstrated the importance of brand using fMRI [6]. Several studies have reported how brands influence consumers’ evaluation of the actual sensory experience [4, 6, 7]. In this paper, we test the hypothesis that the brand effect is observable in a request for choice by considering a protocol where the subject has knowledge about the brand or it is blind to this information, and that we are able to assess this effect by means of physiological signals. In order to do this, we propose an experiment to test the influence of the brand in the expressed preference of a beverage, expanding the analyses of a precedent conference work [8]. The beverage of a well-known brand (Coca-Cola) is tasted and compared with a similar soft drink (Cola), and a request for choice will be performed firstly with unbranded glasses and secondly knowing the name of the two beverages. The consumers’ response is quantified by non-invasive physiological measurements, both through contact and non-contact metrics, such as the SC, the respiratory frequency and the heart rate variability (HRV), being the latter extracted by analysis of face video.

## Materials and methods

### Experimental protocol

The experiment assessed the emotional response elicited by tasting a beverage. In particular, we used the influence of a brand such as Coca-Cola in a request for choice. The study involved human subject research and it was approved by the Politecnico di Milano research ethical committee (comitato etico della ricerca del Politecnico di Milano). Informed written consent was obtained from each participant before starting the experiment. The experiment population comprised of 25 young subjects (13 males) with an age of 20.5 ± 2 years (mean ± standard deviation). The participants were asked to test two beverages, a brand product (Coca-Cola) and a similar soft drink (Cola), the first time without knowing the brand (blind condition, abbreviated to B) and the second time with the knowledge (not blind condition, abbreviated to NB). The volume of the beverage was controlled (around 7 cl) and the glasses were not transparent. After both tastings, they were asked to indicate which beverage they preferred.

The experimental setup comprised an Imaging Source DFK23UM021 camera with 15 mm lens, recording the video of the face of each subject with a framerate of 115 fps and a resolution of 640x480 pixels. The camera was placed in front of the seated subject at a distance of about 1 m [9].

Simultaneously to the video, the SC and respiratory signal were recorded through a FlexComp Infiniti encoder (Thought Technology Inc.). The data were collected using BioGraph Inifiniti software (Thought Technology Inc.) with a sample frequency of 256 Hz and a resolution of 14 bits. The sensors for SC were positioned on the index and ring fingers of the non-dominant hand [10], to make the subject free to grab the beverages. For the respiratory signal, a Velcro belt equipped with a strain gauge was placed on the thorax to perceive the dilation and contraction of the rib cage due to inhalation and exhalation, respectively.

**Fig 1:**
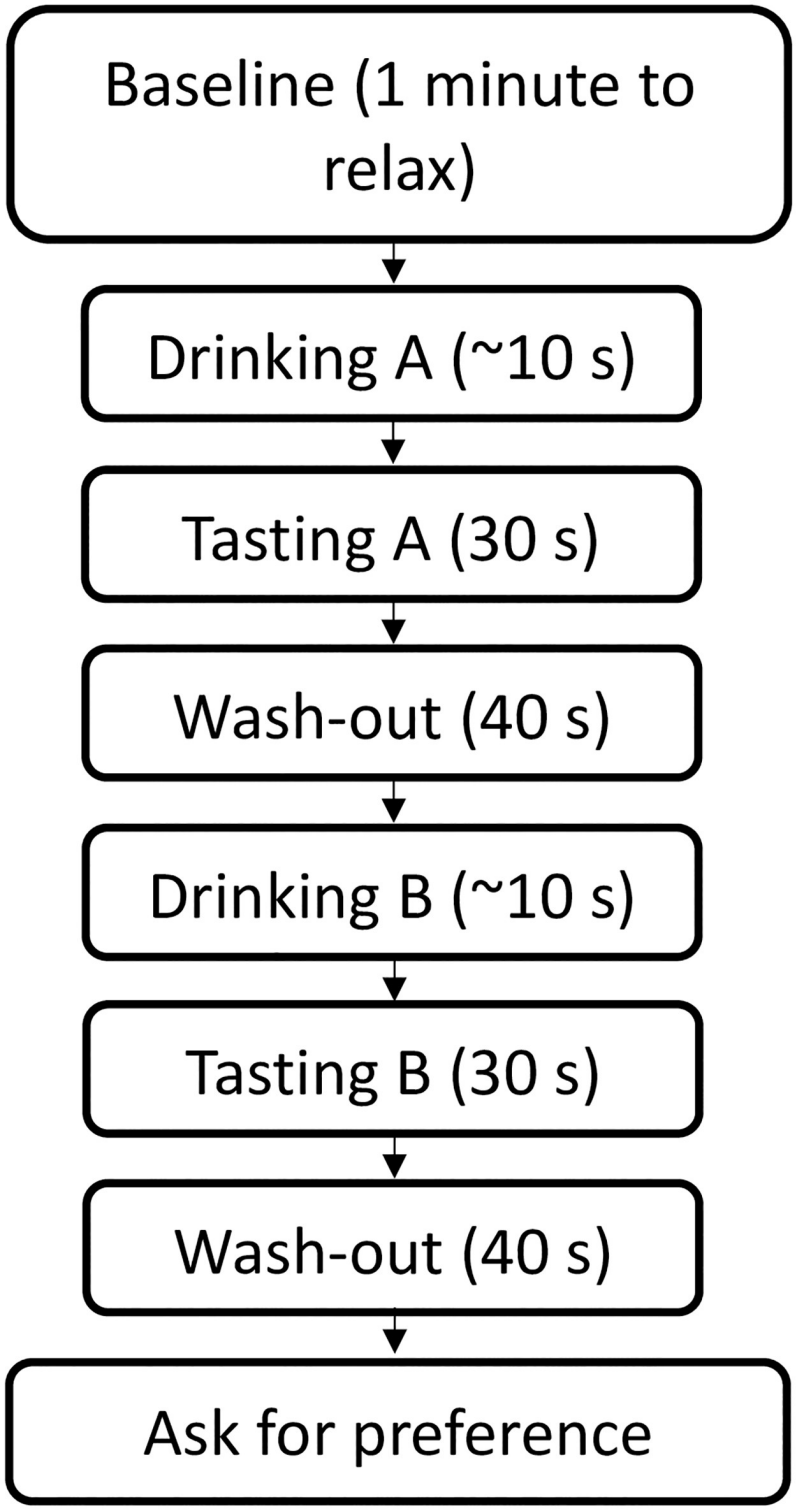
Experimental protocol. Experimental protocol followed in both B and NB condition. After a baseline period during which the subject relaxes, the subject drinks and, after swallowing, tastes the beverage. Then the second beverage is offered, after a wash-out period performed with water. The order of the beverages is random. At the end, the preference between the two soft drinks is asked.

In Fig 1 the experimental protocol for each of the 2 conditions is depicted. A one minute of pre-test period was timed to allow the relaxation of the subject. The physiological signals recorded at the end of this relaxation period were considered the baseline of the subject and the reference for the emotional response assessment. The experimental study consists of one trial per subject split in B and NB conditions. During the B phase, the subject was asked to drink sequentially from two different glasses without knowing the brand of the drink. In the NB phase, on the other hand, the brand of the drink was visible on the glasses. Drinks were given to the subjects in a random order. At the end of each phase the subject was asked to select the preferred beverage. Eventual preference’s changes between the phases were noted. Finally, the average weekly consumption of the Coca-Cola was asked: 8 subjects reported an average/high consumption level of the beverage, whereas 17 declared to rarely or never drink the soft drink. In summary, for our overall analysis the two tests (B and NB) were conducted separately (we stopped the recording after the B test and restarted for the NB). For the first test we considered 25 subjects, whereas for the NB test we considered 24 subjects. For the missing subject in the NB test the camera did not work properly during this part of the experiment. Regarding the preference label, whereas 24 subjects were always able to express a preference, one subject was not able to indicate a preference in neither the B nor NB cases. Therefore, this subject was always considered in the “not preferred” count for both beverages.

### Video analysis

**Fig 2:**
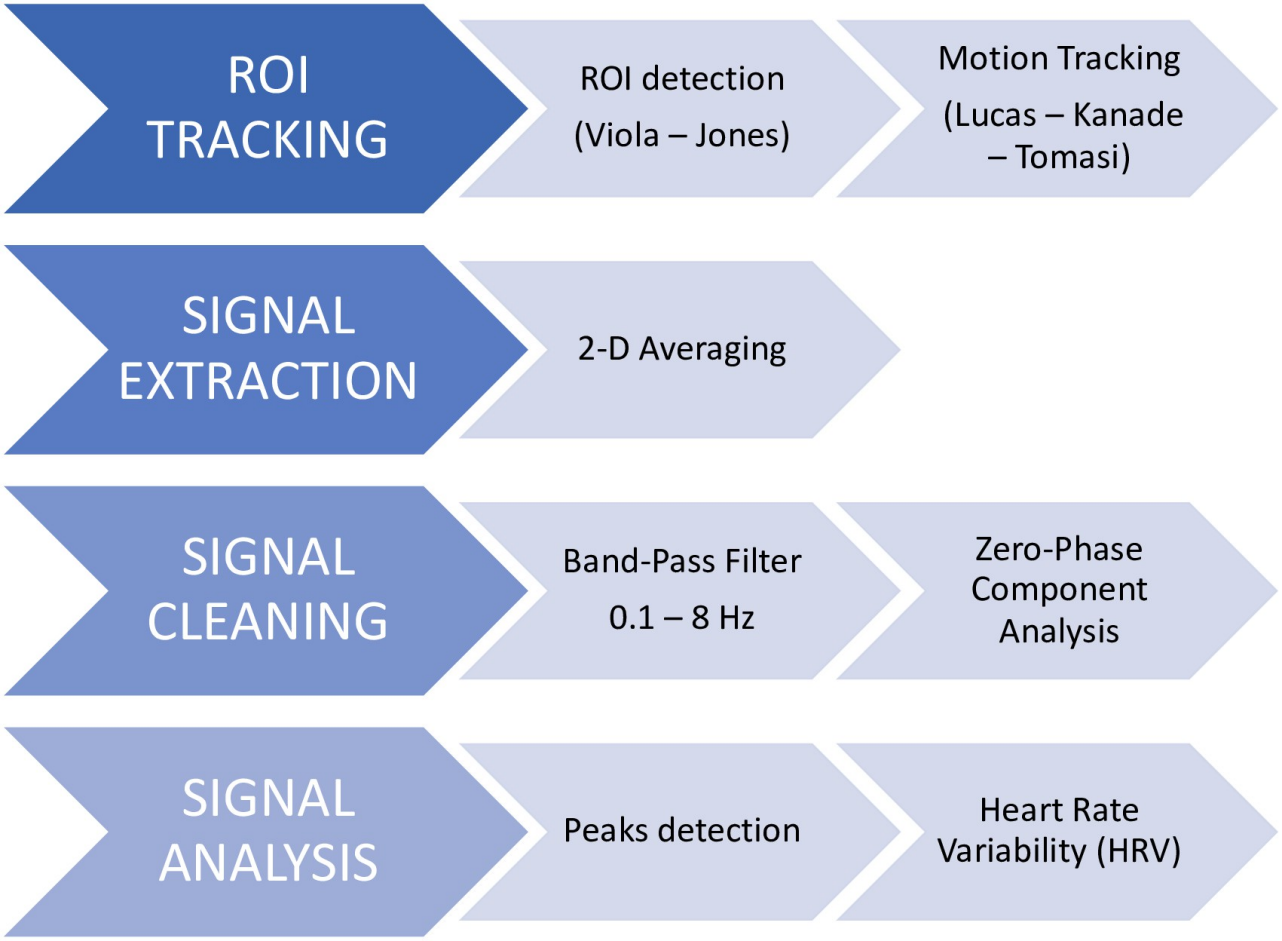
Video processing. In the scheme, the steps applied for the video processing are depicted. Firstly, the ROIs are identified on the face of the subjects and tracked over time. Secondly, a 2-D averaging procedure is applied on the ROIs. Thirdly, a signal cleaning phase follows. Finally, from the thus obtained vPPG signal, the peaks are detected and the HRV parameters computed.

In order to extract the heart rate and HRV metrics, the camera video was processed following the steps depicted in Fig 2 and described in [11]. Three regions of interest (ROIs) were identified by the Viola-Jones face detection algorithm [12] and tracked by means of the Lucas-Kanade-Tomasi algorithm [13]. In particular, the cheek, nose and forehead were selected for the great numbers of blood vessels and the consequent high signal to noise ratio [11, 14, 15].

Secondly, a 2-D averaging procedure was performed in each ROI. After detrending and filtering (Hamming bandpass filter, 0.1–8 Hz) to remove slow head movements and ambient noise, the video-photoplethysmographic signal (vPPG) was extracted through the Zero-Phase Component Analysis method [11]. On the vPPG signal the Automatic-Multiscale Peaks Detection algorithm [16] was applied to detect the systolic points (S). Finally, the following heart rate variability metrics were computed:

- Average peak-to-peak temporal distance (*SS*);
- standard deviation of SS intervals (*std*_*SS*);
- standard deviation of the differences between consecutive SS intervals (*std*_*dSS*);
- percentage of successive interval differences greater than 50 ms (*pNN*50);
- the root mean square of successive differences (*RMSSD*);
- the frequency with highest power in the low frequency band (*LF* *peak freq*, 0.03–0.13 Hz);
- LF power;
- the frequency with highest power in the high frequency band (*HF* *peak freq*, 0.15–0.4 Hz);
- HF power.

In order to assess the changes in cardiac parameters due to the protocol stage, the signals were compared to the individual baseline level through the Eq 1:

$$\begin{array}{c} Index=\frac{Index_{stage}-Index_{baseline}}{Index_{baseline}} \end{array}$$ (1)

where the subscripts identify the considered stage of the protocol.

### Respiratory signal

**Fig 3:**
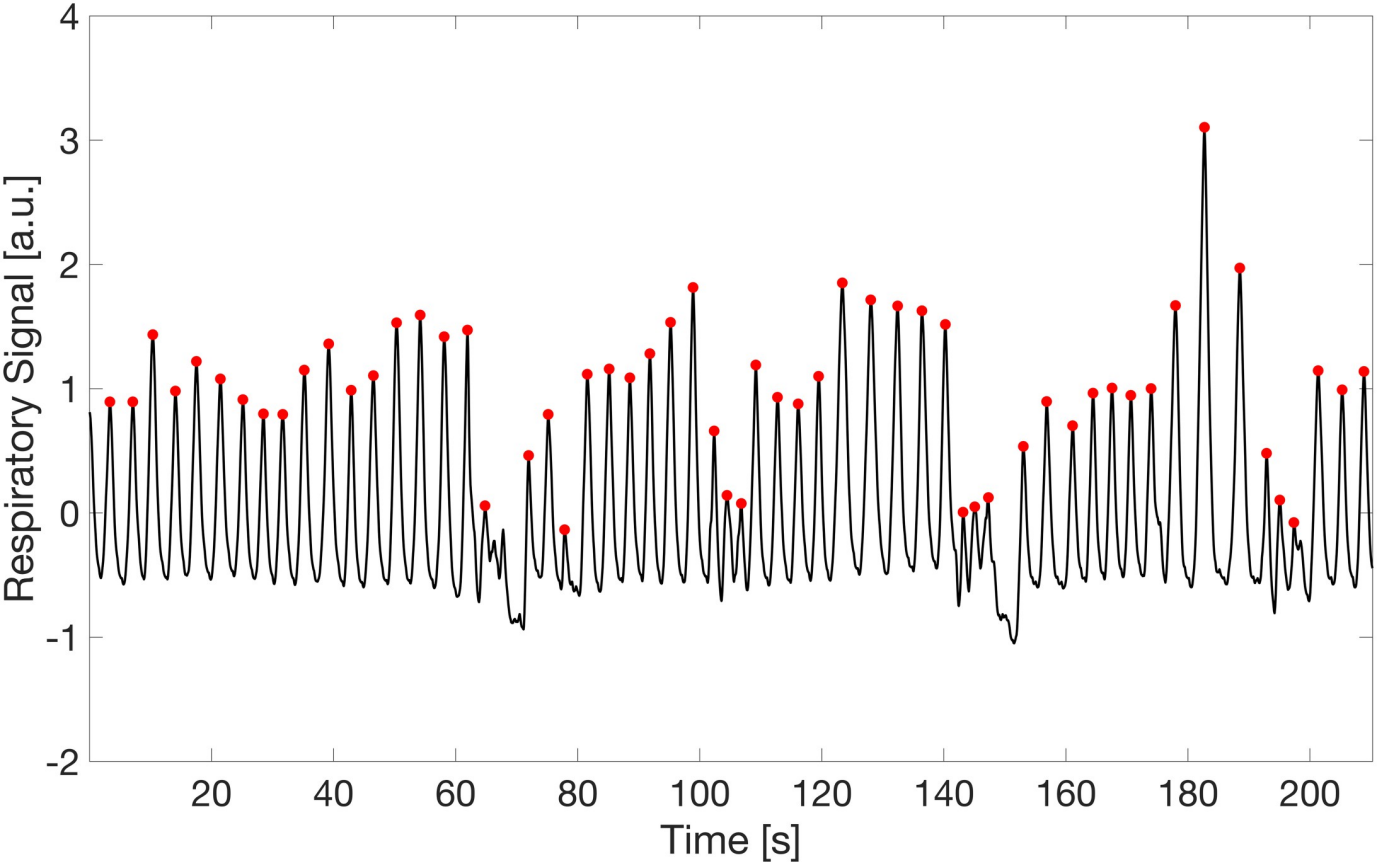
Respiratory signal. The respiratory signal of a participant is depicted. The peaks are marked by red dots. Around the 70th and 150th seconds two deflections are visible, corresponding to the moments in which the participant was swallowing.

Each respiratory peak was found on the respiratory signal as the sample larger than its two neighboring samples, under the condition to be at least 3 seconds from the preceding peak, considering that the normal respiratory rate at rest ranges from 12 to 15 breaths per minute [17, 18]. In Fig 3 an example of a signal is depicted with the corresponding peaks. Interestingly, the swallowing moments are visible in correspondence with the two deflections of the signal, around the 70th and 150th seconds. The average and standard deviation of the distance between consecutive peaks (interbreath interval or *IBI* and *std*_*IBI*, respectively) in the baseline and tasting phases were computed and standardized as in Eq 1.

### Skin conductance analysis

**Fig 4:**
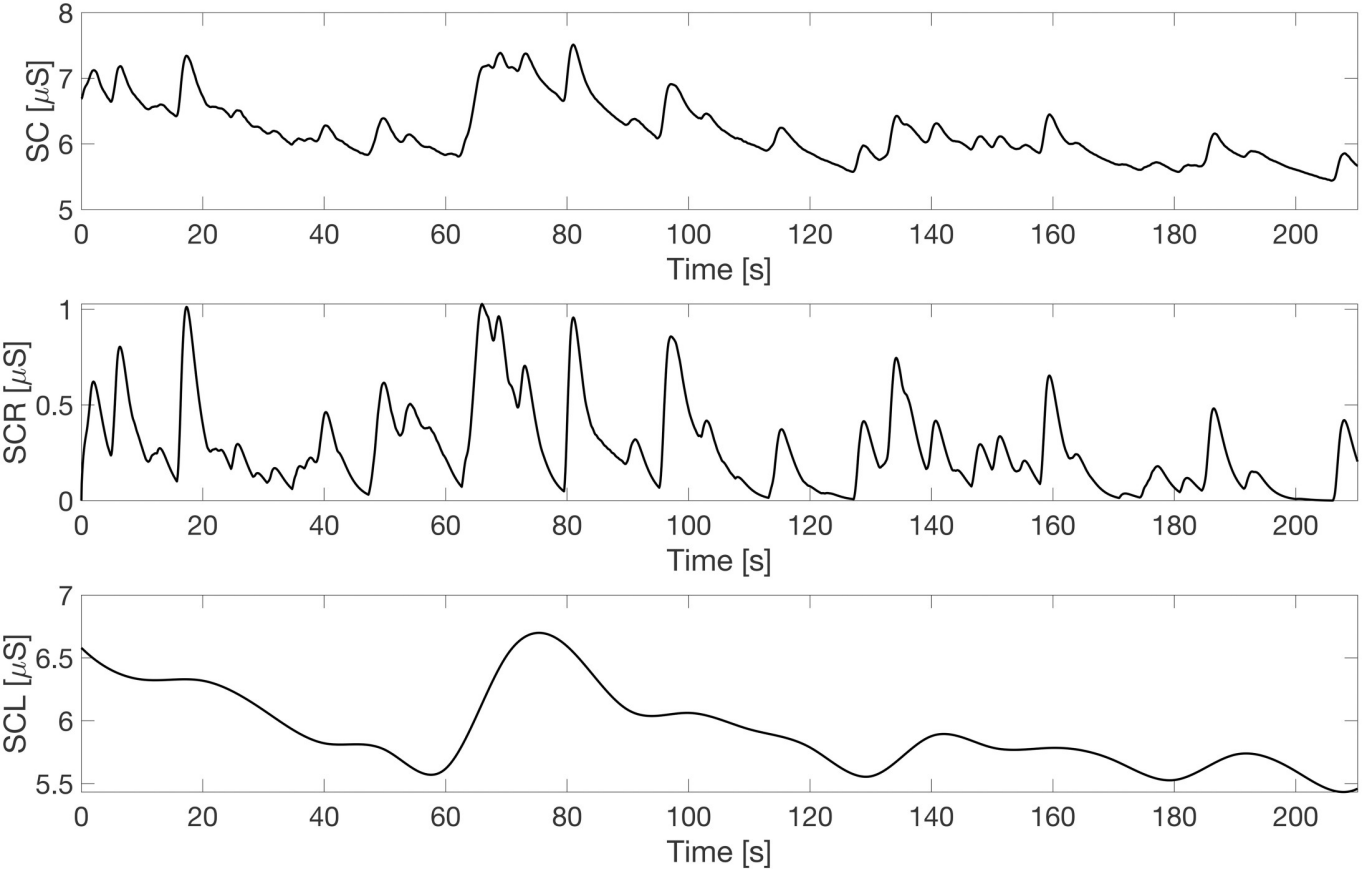
Skin conductance. The SC signal (top panel) is decomposed in its 2 components, the phasic SCR (middle panel) and the tonic SCL (bottom panel).

The SC signal was decomposed in 2 components, the phasic skin conductance response (SCR) and the tonic skin conductance level (SCL), using the cvxEDA algorithm [19]. In Fig 4 the two components are depicted (bottom panels), together with the overall signal (top panel). The SCR measures the effective variation in the signal due to the stimulus response, whereas the SCL gives information on the general level of arousal of the subjects. Finally, the average values of the SC and its two components were computed within each stage of the protocol. In order to have metrics comparable among all subjects, the SC indexes were standardized with the baseline arousal level of the subject, as described in Eq 2:

$$\begin{array}{c} Index=\frac{avg\left(Index_{stage}\right)-avg\left(Index_{baseline}\right)}{avg(Index_{baseline})} \end{array}$$ (2)

where *avg* stands for *average* of the evaluated metric called *Index*. The subscripts identify the considered stage of the protocol.

### Emotional index

The Emotional Index (EI) [20] was computed from the values of SCL and SS previously computed, following the Eqs 3 and 4:

$$\begin{array}{c} EI=1-\beta /\pi \end{array}$$ (3)

$$\begin{array}{c} \beta =\{\begin{array}{cc} \frac{2}{3}\pi +\pi -\theta , & \text{if}\;SCL_{z}\geq 0,SS_{z}\leq 0\text{,}\\ \frac{\pi }{2}-\theta , & \text{otherwise}. \end{array} \end{array}$$ (4)

where *SCL*_*z*_ and *SS*_*z*_ are the Z-scored variables of SS and SCL, respectively, and *θ* is the arctang(*SS*_*z*_, *SCL*_*z*_).

The index is based on the circumplex model of affect plan [21], where the coordinates of a point in space are defined respectively by the heart rate (horizontal axis) to describe the valence and by the SCL (vertical axis) to describe the arousal level. The EI put together the information of both indexes to obtain a monodimenisional variable varying from [-1, 1], where a higher EI value indicates a more positive emotion experienced by the participant [20].

### Statistical analysis

All signals were visually inspected to discard noisy or low quality segment that could compromise the analysis. Furthermore, the outlier values were identified through the interquartile range method and removed. A two-sided Wilcoxon rank sum test was applied for each comparison. Five main comparisons were carried out:

- Blind (B) vs not blind (NB)
  - same beverage group (paired test)
  - when Coca-Cola was preferred
  - when Cola was preferred
- Coca-Cola vs Cola when preferred
- Beverage preferred vs not preferred
- Consumers vs not consumers—all beverages and conditions
- Coca-Cola vs Cola in consumers and not consumers groups.

As an exploratory study, a p-value<0.1 was considered statistically significant. Matlab R2019b (The MathWorks, Natick, MA) was used for all the analyses.

## Results

Quantitative results obtained from the analysis of physiological signals are reported in Tables 1–6. All values are reported as median (interquartile range). In Tables 1 and 2 the significant differences between B and NB conditions are reported. The values were split to perform intra-beverage comparisons (sections 1 and 2 of Table 1) and intra-beverage comparisons according to the preferred soft drink (sections 3 and 4 of Table 2).

**Table 1:** Comparison between B and NB conditions.

Parameters	Blind	Not blind	p-value
	***1—Coca-Cola***		
	N = 24	N = 24	
*SC*	0.01 (0.18)	-0.03 (0.12)	0.679
*SCR*	-0.12 (0.83)	-0.29 (0.85)	0.952
*SCL*	-0.01 (0.12)	-0.04 (0.11)	0.501
*SS*	-0.01 (0.04)	-0.01 (0.02)	0.277
*std_SS*	0.02 (0.24)	-0.11 (0.24)	0.273
*std_dSS*	-0.03 (0.38)	-0.08 (0.42)	0.946
*PNN50*	-0.12 (0.95)	-0.06 (1.63)	0.455
*RMSSD*	-0.03 (0.38)	-0.08 (0.42)	1.000
*LF peak freq*	0.14 (1.33)	0.39 (0.87)	0.250
*LF power*	-0.19 (1.53)	-0.11 (1.42)	0.461
*HF peak freq*	0.08 (0.40)	0.11 (0.40)	0.898
*HF power*	-0.29 (0.75)	-0.27 (0.46)	0.945
*EI*	-0.28 (0.69)	-0.07 (0.82)	0.910
*IBI*	-0.05 (0.24)	0.09 (0.21)	0.588
*std_IBI*	0.17 (0.91)	0.13 (1.28)	0.946
	***2—Cola***		
	N = 24	N = 24	
*SC*	-0.08 (0.16)	-0.00 (0.21)	0.098^+^
*SCR*	-0.47 (0.38)	0.14 (0.86)	0.002*
*SCL*	-0.05 (0.15)	-0.01 (0.18)	0.359
*SS*	0.00 (0.01)	-0.01 (0.04)	0.064^+^
*std_SS*	-0.05 (0.23)	-0.02 (0.24)	0.978
*std_dSS*	0.02 (0.29)	-0.05 (0.41)	0.326
*PNN50*	-0.06 (0.97)	-0.09 (0.90)	0.326
*RMSSD*	0.01 (0.28)	-0.05 (0.41)	0.326
*LF peak freq*	0.22 (1.19)	0.33 (1.66)	0.770
*LF power*	0.23 (1.36)	-0.00 (0.78)	1.000
*HF peak freq*	0.04 (0.51)	-0.11 (0.28)	0.250
*HF power*	-0.30 (0.98)	-0.51 (0.62)	0.313
*EI*	0.00 (0.06)	-0.02 (0.05)	0.557
*IBI*	-0.02 (0.13)	0.02 (0.13)	0.426
*std_IBI*	0.02 (0.74)	0.15 (0.67)	0.903

* p-value<0.05;

^+^ p-value<0.10

**Table 2:** Comparison between B and NB conditions—Second part.

Parameters	Blind	Not blind	p-value
	***3—Preferred Coca-Cola***		
	N = 16	N = 16	
*SC*	0.00 (0.22)	-0.03 (0.09)	0.879
*SCR*	0.12 (0.91)	-0.29 (0.85)	0.473
*SCL*	-0.01 (0.22)	0.00 (0.12)	1.000
*SS*	0.00 (0.04)	-0.01 (0.03)	0.249
*std_SS*	0.07 (0.32)	-0.10 (0.22)	0.095^+^
*std_dSS*	0.09 (0.39)	0.13 (0.64)	0.915
*PNN50*	0.03 (0.878)	0.38 (1.34)	0.460
*RMSSD*	0.09 (0.39)	0.13 (0.63)	0.915
*LF peak freq*	-0.03 (0.78)	0.12 (1.34)	0.485
*LF power*	-0.59 (1.24)	-0.03 (2.13)	0.247
*HF peak freq*	-0.14 (0.32)	-0.05 (0.37)	0.902
*HF power*	-0.39 (1.01)	-0.23 (0.34)	0.628
*EI*	-0.03 (1.09)	-0.06 (0.74)	0.931
*IBI*	-0.01 (0.23)	0.02 (0.20)	0.869
*std_IBI*	-0.26 (0.63)	0.16 (1.00)	0.456
	***4—Preferred Cola***		
	N = 8	N = 7	
*SC*	-0.13 (0.14)	0.11 (0.16)	0.011*
*SCR*	-0.57 (0.36)	0.71 (0.58)	0.003*
*SCL*	-0.12 (0.11)	0.06 (0.14)	0.030*
*SS*	0.00 (0.03)	0.01 (0.01)	0.731
*std_SS*	0.13 (0.40)	-0.01 (0.42)	0.534
*std_dSS*	-0.02 (0.31)	-0.10 (0.36)	0.945
*PNN50*	0.01 (0.94)	-0.32 (0.67)	0.295
*RMSSD*	-0.02 (0.31)	-0.10 (0.36)	0.945
*LF peak freq*	0.80 (0.81)	0.52 (1.49)	0.662
*LF power*	1.19 (1.88)	-0.01 (1.29)	0.052^+^
*HF peak freq*	0.07 (0.22)	-0.11 (0.18)	0.381
*HF power*	-0.02 (0.65)	-0.63 (0.30)	0.064^+^
*EI*	-0.01 (0.09)	-0.43 (0.96)	0.889
*IBI*	-0.06 (0.12)	-0.03 (0.23)	0.329
*std_IBI*	-0.05 (0.56)	0.04 (0.99)	0.524

* p-value<0.05;

^+^ p-value<0.10

**Table 3:** Comparison between Coca-Cola and Cola when preferred.

Parameters	Preferred Coca-Cola	Preferred Cola	p-value
	***B***		
	N = 16	N = 8	
*SC*	0.00 (0.22)	-0.13 (0.14)	0.139
*SCR*	0.12 (0.91)	-0.57 (0.36)	0.027*
*SCL*	-0.01 (0.22)	-0.12 (0.11)	0.200
*SS*	0.00 (0.04)	0.00 (0.03)	0.773
*std_SS*	0.07 (0.32)	0.13 (0.40)	0.669
*std_dSS*	0.09 (0.39)	-0.02 (0.31)	0.536
*PNN50*	0.03 (0.88)	0.01 (0.940)	1.000
*RMSSD*	0.09 (0.39)	-0.02 (0.31)	0.536
*LF peak freq*	-0.03 (0.78)	0.80 (0.81)	0.009*
*LF power*	-0.59 (1.24)	1.19 (1.88)	0.017*
*HF peak freq*	-0.14 (0.32)	0.07 (0.22)	0.445
*HF power*	-0.39 (1.01)	-0.02 (0.65)	0.429
*EI*	-0.03 (1.09)	-0.01 (0.09)	1.000
*IBI*	-0.01 (0.23)	-0.06 (0.12)	0.213
*std_IBI*	-0.26 (0.63)	-0.05 (0.56)	0.847
	***NB***		
	N = 16	N = 7	
*SC*	-0.03 (0.093)	0.11 (0.16)	0.145
*SCR*	-0.29 (0.85)	0.71 (0.58)	0.040*
*SCL*	0.00 (0.12)	0.06 (0.14)	0.267
*SS*	-0.01 (0.03)	0.01 (0.01)	0.264
*std_SS*	-0.10 (0.22)	-0.01 (0.42)	0.955
*std_dSS*	0.13 (0.64)	-0.10 (0.36)	0.388
*PNN50*	0.38 (1.34)	-0.32 (0.67)	0.118
*RMSSD*	0.13 (0.63)	-0.10 (0.36)	0.388
*LF peak freq*	0.12 (1.34)	0.52 (1.49)	0.429
*LF power*	-0.03 (2.13)	-0.01 (1.29)	0.662
*HF peak freq*	-0.05 (0.37)	-0.11 (0.18)	0.833
*HF power*	-0.23 (0.34)	-0.63 (0.30)	0.024*
*EI*	-0.06 (0.74)	-0.43 (0.96)	0.857
*IBI*	0.02 (0.20)	-0.03 (0.23)	0.768
*std_IBI*	0.16 (1.00)	0.04 (0.99)	0.797

* p-value<0.05;

^+^ p-value<0.10

**Table 4:** Comparison of a beverage when preferred/not preferred.

Parameters	Preferred	Not Preferred	p-value
	***Coca-Cola***		
	N = 32	N = 17	
*SC*	-0.02 (0.16)	-0.01 (0.15)	0.960
*SCR*	-0.11 (0.85)	-0.32 (0.87)	0.179
*SCL*	0.00 (0.15)	-0.01 (0.06)	0.907
*SS*	-0.01 (0.04)	-0.02 (0.03)	0.098^+^
*std_SS*	-0.01 (0.30)	-0.07 (0.24)	0.225
*std*_*dSS*	0.13 (0.44)	-0.12 (0.28)	0.045*
*PNN50*	0.10 (0.98)	-0.32 (0.69)	0.162
*RMSSD*	0.13 (0.43)	-0.12 (0.27)	0.049*
*LF peak freq*	-0.03 (0.87)	0.41 (0.87)	0.164
*LF power*	-0.35 (1.48)	-0.11 (1.38)	0.524
*HF peak freq*	-0.10 (0.38)	0.14 (0.40)	0.041*
*HF power*	-0.31 (0.52)	-0.26 (0.53)	0.720
*EI*	-0.03 (0.88)	-0.28 (0.59)	0.602
*IBI*	0.01 (0.21)	-0.06 (0.26)	0.224
*std_IBI*	0.10 (0.75)	0.25 (1.56)	0.890
	***Cola***		
	N = 15	N = 34	
*SC*	-0.09 (0.19)	-0.02 (0.15)	0.597
*SCR*	-0.39 (1.33)	-0.18 (0.63)	0.887
*SCL*	-0.08 (0.19)	-0.02 (0.13)	0.501
*SS*	0.00 (0.02)	0.00 (0.02)	0.301
*std_SS*	0.09 (0.40)	-0.02 (0.18)	0.818
*std_dSS*	-0.02 (0.33)	0.02 (0.33)	0.469
*PNN50*	-0.09 (0.73)	-0.06 (1.03)	0.356
*RMSSD*	-0.02 (0.33)	0.01 (0.33)	0.489
*LF peak freq*	0.52 (0.93)	0.01 (1.37)	0.111
*LF power*	0.44 (2.03)	-0.27 (0.74)	0.016*
*HF peak freq*	0.03 (0.27)	-0.05 (0.45)	0.409
*HF power*	-0.50 (0.61)	-0.51 (0.88)	0.817
*EI*	-0.01 (0.32)	-0.01 (0.05)	1.000
*IBI*	-0.04 (0.16)	0.01 (0.12)	0.327
*std_IBI*	0.04 (0.72)	0.16 (0.63)	0.430

* p-value<0.05;

^+^ p-value<0.10

**Table 5:** Comparison between Coca-Cola consumers and not consumers.

Parameters	Consumers	Not consumers	p-value
	N = 32	N = 66	
*SC*	-0.08 (0.16)	-0.02 (0.13)	0.257
*SCR*	-0.37 (1.06)	-0.15 (0.81)	0.086^+^
*SCL*	-0.03 (0.17)	-0.01 (0.11)	0.728
*SS*	0.00 (0.03)	-0.01 (0.03)	0.120
*std_SS*	-0.04 (0.22)	-0.02 (0.26)	0.673
*std_dSS*	0.00 (0.31)	-0.03 (0.40)	0.723
*pNN50*	0.19 (1.28)	-0.11 (1.03)	0.089^+^
*RMSSD*	0.00 (0.31)	-0.03 (0.40)	0.714
*LF peak freq*	0.25 (1.24)	0.22 (0.98)	0.368
*LF power*	-0.36 (0.94)	0.14 (1.44)	0.076^+^
*HF peak freq*	-0.02 (0.42)	0.05 (0.35)	0.813
*HF power*	-0.26 (0.68)	-0.41 (0.64)	0.682
*EI*	0.00 (0.11)	-0.07 (0.71)	0.045*
*IBI*	-0.06 (0.18)	-0.01 (0.18)	0.480
*std_IBI*	0.12 (0.74)	0.08 (0.98)	0.580

* p-value<0.05;

^+^ p-value<0.10

**Table 6:** Comparison of the beverages in the consumers/not consumers groups.

Parameters	Coca-Cola	Cola	p-value
	***Consumers***		
	N = 16	N = 16	
*SC*	-0.04 (0.15)	-0.10 (0.25)	0.371
*SCR*	-0.46 (1.07)	-0.35 (1.04)	0.740
*SCL*	-0.02 (0.13)	-0.06 (0.23)	0.566
*SS*	0.00 (0.04)	0.00 (0.02)	0.696
*std_SS*	-0.04 (0.21)	-0.02 (0.31)	0.865
*std_dSS*	0.08 (0.46)	-0.03 (0.25)	0.765
*PNN50*	0.34 (1.76)	0.07 (0.85)	0.662
*RMSSD*	0.08 (0.46)	-0.03 (0.25)	0.765
*LF freq peak*	0.06 (0.93)	0.25 (1.45)	0.400
*LF power*	-0.36 (0.63)	-0.31 (1.01)	0.667
*HF freq peak*	0.06 (0.38)	-0.04 (0.45)	0.910
*HF power*	-0.23 (0.42)	-0.72 (0.65)	0.200
*EI*	-0.03 (0.63)	0.00 (0.04)	0.805
*IBI*	0.03 (0.24)	-0.06 (0.09)	0.340
*std_IBI*	0.17 (0.69)	-0.04 (0.60)	0.838
	***Not Consumers***		
	N = 33	N = 33	
*SC*	-0.01 (0.13)	-0.02 (0.13)	0.927
*SCR*	-0.12 (0.82)	-0.18 (0.80)	0.612
*SCL*	-0.01 (0.10)	-0.02 (0.11)	0.99
*SS*	-0.01 (0.02)	0.00 (0.02)	0.043*
*std_SS*	-0.01 (0.33)	-0.04 (0.22)	0.855
*std_dSS*	-0.04 (0.33)	0.02 (0.43)	0.629
*PNN50*	-0.11 (0.75)	-0.17 (1.18)	0.961
*RMSSD*	-0.04 (0.33)	0.01 (0.43)	0.629
*LF freq peak*	0.32 (0.85)	0.17 (1.32)	0.891
*LF power*	0.78 (1.57)	0.05 (0.85)	0.538
*HF freq peak*	0.09 (0.57)	0.01 (0.42)	0.075^+^
*HF power*	-0.37 (0.51)	-0.47 (0.71)	0.874
*EI*	-0.45 (0.70)	-0.01 (0.28)	0.036*
*IBI*	-0.03 (0.22)	0.01 (0.12)	0.691
*std_IBI*	-0.07 (1.33)	0.11 (0.84)	0.554

* p-value<0.05;

^+^ p-value<0.10

In Table 3, the metrics that showed significant differences between the two beverages when preferred are reported, whereas in Table 4 the results of the comparison between the trials in which a beverage was preferred or not were reported. Finally, in Table 5 the comparisons of the physiological indexes between consumers and not consumers are showed, whereas in Table 6 the comparisons of the beverages in the two groups are reported.

**Product preference analysis**: Among the 25 subjects, 11 (44%) changed their preference after they tried the branded beverage: six subjects that chose the Cola in the B condition changed their preference to Coca-Cola when the brand was known. Furthermore, 5 subjects that chose Coca-Cola in the first phase, changed their preference to Cola in the NB phase.

**Blind vs not blind analysis**: The parameters related to the SC were higher in the NB phase (section 2 of Table 1 and section 4 of Table 2), whereas the *std*_*SS* and *SS* were lower (section 2 of Table 1 and section 3 of Table 2).

After the comparison between phases, the physiological response elicited by the two beverages when preferred were analyzed in the two conditions. The soft drink with highest arousal changed between the 2 conditions: during the B phase, the SCR was higher and both LF and its power were lower for Coca-Cola, whereas in the NB phase the SCR was higher and the HF power lower for Cola (Table 3).

**Physiological response analysis based on preference**: Considering together both blind and not blind conditions, additional analyses were performed for the first time. The comparison of the physiological status when a beverage was preferred or not showed no significant difference when the beverages were considered together, but different results were found splitting in accordance to the brand: when preferred, higher HRV and lower HF peak frequency were found when the drink was Coca-Cola and higher LF when Cola was considered (Table 4).

**Physiological response analysis based on consumption**: Finally, we compared the physiological responses of the population that declared to have an average/high level of consumption of Coca-Cola (consumers) and the population that rarely or never drinks the famous soft drink (not consumers). It emerged a lower SCR for consumers together with higher pNN50, lower LF power and higher EI (Table 5). Comparing Coca Cola and Cola in the 2 groups, no significative difference emerged in the consumer group, whereas in the not consumers it was found lower SS, higher HF peak frequency and lower EI for Coca-Cola (Table 6).

## Discussion

It has been observed that the brand name influences the way a consumer experiences the taste of a product [4, 6]. Joubert et al. tested 312 participants who evaluated milk from various milk brands in either a branded or unbranded packaging. They found that the brand name can influence consumers’ evaluation of the actual sensory taste for milk products [4]. In particular, they found the once the milk brand was revealed, the product of the dominant market brand increased its liking rating, whereas the products of smaller brands obtained lower scores than before. All these assessments were however conducted using face-to-face interviews, thus introducing a human, subjective component that can bias the responses [22]. Indeed, the use of interviews or questionnaires lead to the introduction of a rationalization phase between the product’s experience and the evaluation, and adding also a social pressure in being potentially subject to the interviewer judgment. McClure at al., instead, chose to evaluate the effect of brand using the physiological responses elicited in the subjects, thus filtering any possible rationalization phase. McClure compared Coca-Cola to Pepsi measuring the cerebral activation through functional Magnetic Resonance Imaging (fMRI) in a tasting comparison. It was found that in a blind condition there was a similar preference between the two beverages. However, when the beverage was labeled as Coca-Cola, it was preferred significantly more that when it was not labeled or when the cup was labeled as Pepsi. In the fMRI, this resulted in the activation of different areas of the brain [6].

In this study, alternative ways to evaluate the emotional components in consumer preferences have been assessed using the SC, the HRV and the respiratory signal, thus implying less invasive and expensive experimental setups.

Skin conductance has been associated with the activity of sweat glands innervated by the sympathetic branch of the autonomic nervous system and it is often used as a measure of arousal [23]. Garczarek-Ba̧k et al. proved that the SC could give useful insights about video advertisement effectiveness. Indeed they found a significant correlation between the number of peaks in the SC recorded during a spot and the amount of chosen products [24]. Vila-López and Küster-Boluda proved that lower electrodermal activity is present during exposure to the packaging that will be chosen in a request for choice [22] and Walla et al. found that “skin conductance was significantly reduced in case of viewing liked brand names compared to viewing disliked brand names” [25]. In the first case, Vila-López and Küster-Boluda exposed 83 participants to 6 different packagings, recording their SC as unconscious response and their verbal opinion about product choice as conscious response. They observed that the most often selected packaging had lower SC [22]. Similarly, in the study by Walla et al., 29 subjects were exposed to different brands that were precedently rated in a liking scale. The 10 most liked and the 10 most disliked brands in a list of 300 brands were selected for each participant. The authors reported a significantly reduced SC associated with visual presentations of liked brand names compared to the disliked ones.

HRV has also been used to recognize emotional states [26]. In the same study by Walla et al. precedently cited, they also found that a “strong trend towards an increase in heart rate during visual presentation of known disliked brand names” [25]. In the last years, methods to measure HRV through contactless technology have arisen, in order to reduce the possible effect of the measurement system on the emotional response of the subjects [3].

Features extracted from the respiratory signal have been used in the literature to classify emotions [27, 28]; moreover, respiratory changes have been associated to different cognitive loads [29]. In this study the respiratory rate was assessed, but no significance was found for any comparison.

In our study, our hypothesis that there is an effect on the emotional response knowing the brand during product’s consumption was proved by: 1) the significant difference found in the skin conductance parameters between the two preferred beverages in both experimental conditions; 2) the changes of preference observed from the B to the NB condition; 3) the changes in physiological response obtained by comparing the usual consumers of Coca-Cola and the subjects that rarely or never drink the beverage; 4) the differences between the 2 soft drinks observed comparing the physiological response recorded when a beverage was later chosen as preferred or not.

Regarding the first point, while in the B condition the SCR values were higher for the Coca-Cola and both the LF peak frequency and its power were lower, in the NB condition the SCR was higher for the not famous brand, probably due to a surprise factor, as confirmed by the lower HF power (see Table 3). The changes in the other contactless metrics were not sufficient to highlight differences between brands, but they were sensitive to B vs. NB conditions. Both *SS* and *std*_*SS* were higher and the SC features lower in the B condition, suggesting a lower emotional load [30]. Coherently, the HF power was lower in the NB condition (Table 2, section 4) and the SCR higher, suggesting a higher emotional load [30]. Furthermore, the lower SC metrics in the blind condition is in agreement with a study by Smith et al. that found a significant increase in children’s arousal when viewing branded beverage products compared to their unpackaged counterparts [31].

Regarding the second point, 11 subjects (44%) changed their preference after they tried the beverages knowing the brands. The 6 subjects (24%) that changed preference from Cola to Coca-Cola provide evidence that the knowledge of the brand affects subject’s choice. The 5 subjects that changed from Coca-Cola in B condition to Cola in NB conditions were not habitual consumers of soft drinks in general. A possible explanation of the change resides in the fact that, not being attached to the brand name, they probably wanted to step back from the icon of the most known soft drink.

Another finding highlighting the difference between the usual consumers of Coca-Cola and the population that rarely or never consume the soft drink was the different physiological response measured in the 2 cases (see Table 5): the lower SCR observed in the consumers suggest a more relaxed approach of the usual consumers of the beverage to the test, that, together with the higher pNN50 and lower LF power, points to a lower emotional load. Furthermore, the EI, even presenting values very close to 0 in both cases, was significantly higher in the consumer group, as it could be expected. To our knowledge, this is the first time that the EI has been computed to evaluate the strength of a brand in a tasting experiment. Similar findings were noted comparing the two beverages in the consumers/not consumers groups: we noted that there was no significant difference between the two beverages in the first group, that was in general relaxed (low skin conductance parameters), whereas in the not consumers group the higher SS, lower HF peak and higher EI for the Cola point towards a lower emotional load for that beverage (see Table 6).

Regarding the last point, it is interesting to note that no index showed a significant difference comparing the trials in which the beverages were later chosen as preferred and the trials in which the beverages were not chosen. However, considering the two beverages separately, we obtained opposite results based on the soft drink considered (see Table 4): for Cola there was a higher LF power when preferred, whereas for Coca-Cola a general higher level of activation when preferred was observed. Indeed for Coca-Cola there was higher HRV and a lower HF peak frequency, linked to a lower respiratory frequency, as confirmed by the higher interval between respiratory peaks observed in this case (0.01 ± 0.21 for Coca-Cola when preferred, vs -0.06 ± 0.26 when Coca-Cola was not chosen, p = 0.224), even if this result was not significant. Further studies are needed to better interpret this result.

The main limitation of the study was the low number of subjects, all young Italians of similar socio-economic background and lifestyle. The number of Coca-Cola consumers/not consumers was biased in favor of the first group and the weekly consumption of other soft drink was not investigated. Moreover, in order to assess the effect of brand on product choice and on physiological reactions, different brands should be considered in possibly different fields. In future studies, a larger and more heterogeneous population could be selected, with the possibility to assess consumers behaviors in different age, sex and cultural subgroups.

## Conclusion

The proposed study focused on the evaluation of consumer preference by means of physiological signals. In particular, a comparison between the well-known Coca-Cola beverage and a similar soft drink was performed in both blind and not blind conditions. The preferred beverage was asked at the end of each test. In summary:

### Main results

We demonstrated that a brand effect is present, as assessed by skin conductance parameters between the 2 preferred beverages in both experimental conditions and the change of preference by 44% of the subjects. Moreover, the effect of the brand was visible by comparing the physiological responses when a beverage was chosen as preferred or not. Indeed, while aggregating both beverages in the analysis no parameter showed a significant difference in the two cases, splitting in accordance with the beverage highlighted an opposite, brand-related behavior.

### Contribution to the field

The physiological results and the changes of preference observed between the two phases show that knowing the brand of a popular product can have an immediate emotional response. As a consequence of these findings, future marketing related studies and protocols should be performed taking into consideration that knowing the brands can influence even the first emotional response.

### Future studies

Similar studies should be done considering the effects of different brands in different fields, possibly considering also other senses (such as smell and touch). Furthermore, further studies should be done considering a more heterogeneous population.

In conclusion, this study paves the way to a better understanding of the brand mechanisms behind brand conditioning and the bias that brands introduce in the consumer’s experience and preference as well as, more in general, to a more quantitative characterization of any subjective behavioral bias from the surrounding physical and social environment.

## Supporting information

S1 Data (XLSX)
